# Climate change and child health in Europe: a scoping review of emerging impacts

**DOI:** 10.1007/s00431-026-06966-8

**Published:** 2026-05-05

**Authors:** Sarah van den Berg, Daan van der Stadt, Shahriyar Shahbazi Khamas, Fleur Sondaal, Faridi Jamaludin, Lieke van Baardewijk, Susanne Vijverberg, Berber Kapitein

**Affiliations:** 1 Pediatric Intensive Care Unit, Emma Children’s Hospital, AmsterdamUniversity Medical Center, Amsterdam, The Netherlands; 2 Vrije Universiteit Amsterdam, Amsterdam, The Netherlands; 3 Department of Pulmonary Medicine, Amsterdam University Medical Center, University of Amsterdam, Amsterdam, The Netherlands; 4 Medical Library, Amsterdam University Medical Centre, University of Amsterdam, Amsterdam, The Netherlands; 5 Amsterdam University Medical Center, University of Amsterdam, Amsterdam, The Netherlands; 6 Department of Public Health, Julius Center for Health Sciences and Primary Care, University Medical Center Utrecht, Utrecht, The Netherlands; 7 Department of Pediatric Pulmonology and Allergy, Emma Children’s Hospital, Amsterdam University Medical Center, Amsterdam, The Netherlands

**Keywords:** Climate change, Child health, Europe

## Abstract

Children are extremely vulnerable to climate-related environmental impacts and air pollution due to both biological and behavioral factors. Despite the emerging evidence of the increasing effects of climate change on the world and on children’s health, policies to drive change and halt the effects are lacking. Climate change is causing Europe to heat up faster than other continents. Here, we assessed the evidence-based effects of climate change and air pollution on child health in Europe. A scoping review was performed to map the impact of climate-related exposures on the health of children in Europe. A literature search was conducted in three bibliographic databases (PubMed, Embase.com, and Cochrane Database of Systematic Reviews/Cochrane Central Register of Controlled Trials), for studies published between January 1, 2014, and November 11, 2024. Studies were included if they met the following criteria: original studies performed in Europe, addressing climate-related exposures (i.e., the effects of air pollution, heat stress and/or wildfires) on clinical outcomes in neonates, infants, and children (<18 years). The literature search generated a total of 3838 unique articles; upon screening, 73 articles were included in this scoping review. Most studies were conducted in South and West Europe. Climate-related exposures were linked to negative neonatal outcomes, increased risk of respiratory and allergic disease, adverse neurological development, and a higher incidence of metabolic conditions in children. Most studies assessed the impact of air pollution (mainly particulate matter with a diameter smaller than 2.5 micrometers PM_2.5_ and PM_10_ exposure); few studies assessed other climate-related outcomes such as heat stress or wildfires.

*Conclusion*: Climate change is an active driver of pediatric morbidity in Europe, posing urgent respiratory, neurological, and perinatal risks amplified by social inequality. Protecting future generations demands a paradigm shift in healthcare that moves beyond treating acute symptoms to addressing upstream environmental drivers, including the integration of environmental exposure data into clinical practice.

**Table:** 

**What is Known:** • *Children are biologically vulnerable to environmental hazards and more prone to climate-related exposures.* • *Europe is warming up faster than other continents; therefore, children in Europe are at increased risk of climate-related adverse health outcomes.*
**What is New:** • *This scoping review confirms climate change may lead to increased pediatric morbidity in Europe, linking air pollution, heat stress, and wildfires to perinatal, respiratory, metabolic, and neurological risks.* • *European studies regarding climate change and children’s health have mainly focused on the impact of air pollution, but hardly focused on the impact of heat stress, highlighting the need for broader research and a coordinated pan-European change in political interventions tackling climate change.*

**Supplementary Information:**

The online version contains supplementary material available at 10.1007/s00431-026-06966-8.

## Introduction

The combustion of fossil fuels is a major source of air pollution and greenhouse-gas emissions, and therefore a driver of climate change. This combustion releases large amounts of fine, respirable particles (e.g. particulate matter (PM)), nitrogen dioxide (NO_2_), sulfur dioxide (SO_2_), polycyclic hydrocarbons, and volatile chemicals, forming ground-level ozone (O_3_) [1]. The emission of these air pollutants has risen sharply in the past 70 years. As a result, the average surface temperature of the earth has increased by approximately 1.5 °C since preindustrial times [2]. Especially in Europe, this is a pressing problem, where the temperature is rising twice as quickly as compared to the rest of the world, which is also reflected in the increase in wildfire activity during the summer of 2025 with wildfires in Portugal, Spain, southern France, southern Italy, Greece, Turkey, Ukraine, and parts of the UK [3, 4].

Climate change challenges children’s right to survival, good health, well-being, and nutrition [5]. Children are extremely vulnerable to climate-related environmental impacts due to both biological and behavioral factors. Not only prolonged exposure to environmental toxic effects but also the development and growth of their organ systems and immune systems make children highly susceptible to the detrimental effects of toxic chemicals and other stressors. Since defense systems such as the immune systems, DNA repair systems, and other mechanisms for detoxifying chemicals are still under development, children are more predisposed to physical toxicants and psychological stress [1].

Climate-related exposures interact with existing social and structural inequities. Studies show that minority and socioeconomically disadvantaged children are disproportionately exposed to higher levels of air pollution [6], illustrating that climate change and environmental injustice are deeply intertwined. Despite the emerging evidence, policies to drive change and halt the harmful effects are lacking [3, 7]. Europe has been reported to be the fastest-warming continent [8]. However, a comprehensive overview of climate-related health effects on children in Europe is not available and could help identify knowledge gaps and guide future research, policy development, and practices [9]. Therefore, we performed a scoping review of existing literature on the effects of climate change on child health specifically in Europe.

## Methods

### Statement

A scoping review method was selected due to the nature of the research question and the wide range of studies addressing this question [10, 11]. The conduct and reporting of this review adhere to the established principles of the PRISMA extension for scoping reviews [12]. Air pollution can be seen as a driver of climate change, leading to increased temperatures and resulting in wildfires. All of these factors may have harmful health effects in children; therefore, in this review, these events are defined as “climate change.”

### Search

A literature search was performed in three bibliographic databases (PubMed, Embase.com, and Cochrane Database of Systematic Reviews/Cochrane Central Register of Controlled Trials) from inception to November 11, 2024. Searches were performed by a medical information specialist (FJ). Duplicate articles were excluded using the R-package “ASYSD,” an automated deduplication tool, followed by manual deduplication in Endnote (X20.0.3). The full search strategy used for each database is detailed in appendix A in the supplementary material. Search terms (including synonyms), closely related words and keywords used as in the Medical Subject Heading (MeSH) index or free-text words were “climate change”, “child health”, “global health”, and “Europe.”.

### Selection process and data assessment

To be included in the review, studies had to be original studies in English with the research executed in Europe. Subjects in the studies needed to be children under the age of 18 or presenting age-stratified results. The focus of the studies had to be climate change, i.e., the effects of air pollution: either air pollution solely, increased temperature solely, wildfires solely, or a combination on health outcomes in children in Europe. In addition, studies were excluded if they were of the following publication types: reviews, editorials, letters, legal cases, or interviews. Two reviewers (DS and BK) independently screened all potentially relevant titles and abstracts for eligibility using Rayyan [13]. Discrepancies in judgement regarding inclusion were discussed by SB, DS, LB, and BK and reached through a consensus procedure. Since the past decade has shown an enormous rise in temperatures and amount of wildfires, it was decided to focus on papers published after Jan 1, 2014.

### Role of funding source

No external funding was obtained for this work.

## Results

### Identification and characteristics of included studies

**Fig. 1:**
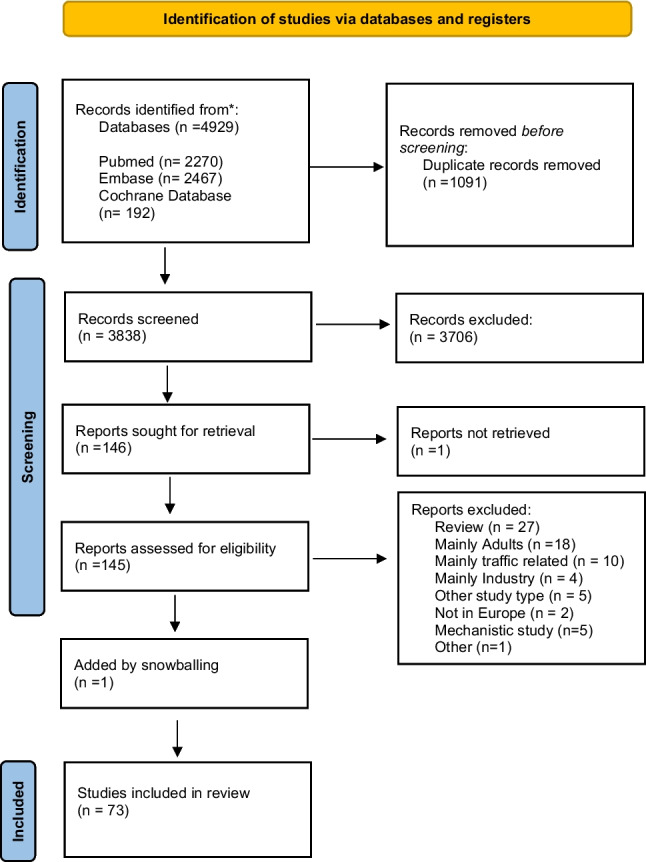
Flowchart of identified articles and articles included in the review

**Fig. 2:**
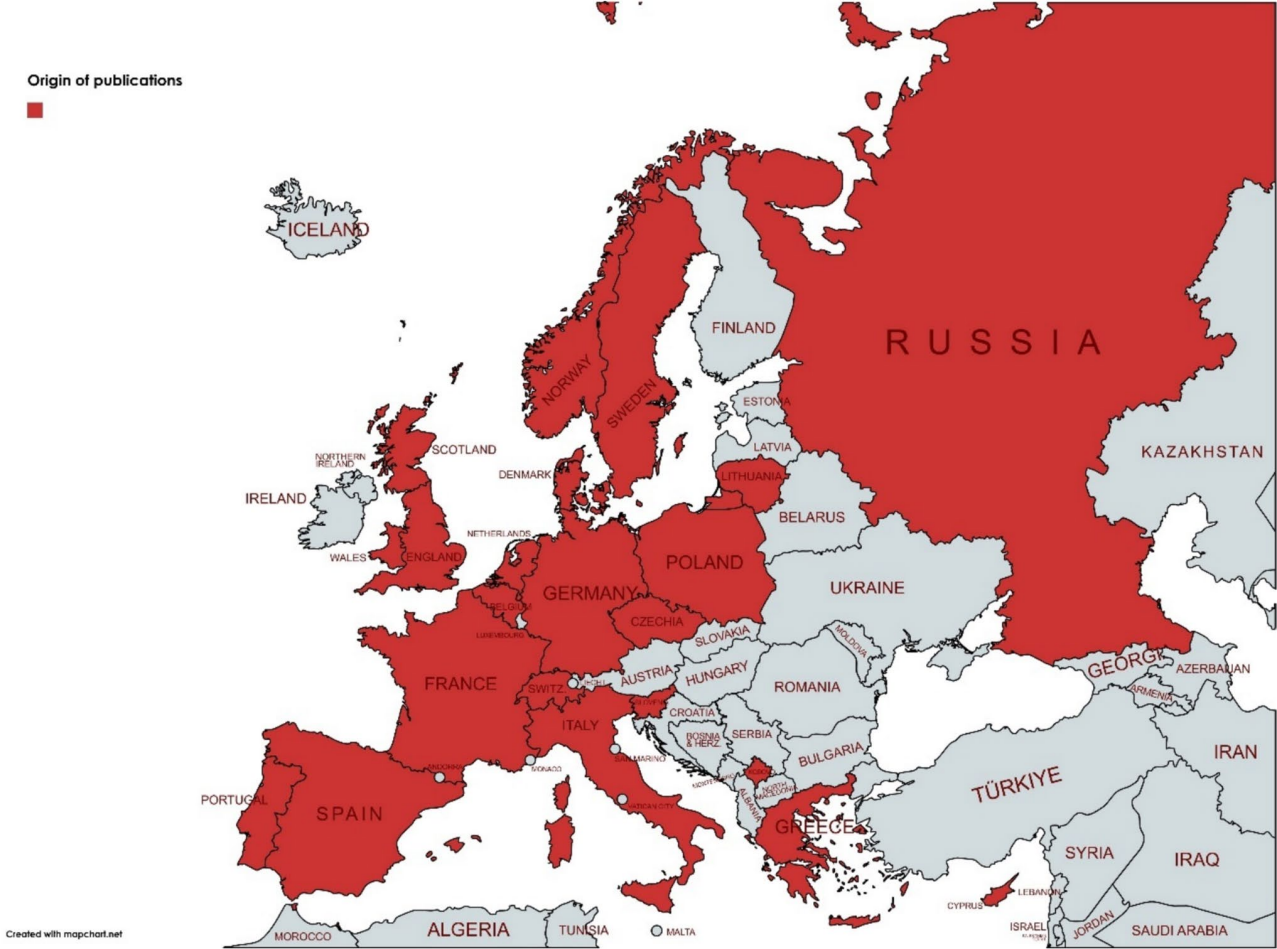
Origin of publications (©Europe | MapChart)

The literature search generated a total of 4929 references: 2270 in PubMed, 2467 in Embase.com, and 192 in the Cochrane Databases. After removing duplicates of references, 3838 unique references remained. After screening titles and abstracts, the full text of 146 articles was read. A flow chart of the search and selection process is presented in Fig. 1. In total, 73 studies were included in this review. Table 1 provides a comprehensive summary of the study characteristics included in this review. Studies on the effects of climate change on European children are published across the continent; see Fig. 2. The primary climate-related health impacts that have been mainly described are related to air pollution (92%); other studies described the effects of heat stress (5%) or wildfires (3%). A summary of the outcomes of the included studies is described in Supplementary Table 1.

**Table 1:** Study characteristics of the included studies

Study characteristics	Air pollution (*n*, %)	Heat stress (*n*, %)	Wildfires (*n*, %)
**Number of studies**	*67 (100%)*	*4 (100%)*	*2 (100%)*
Country of origin			
North Europe	11 (16%)		
East Europe	6 (9%)		
South Europe	25 (37%)	3 (75%)	2 (100%)
West Europe	20 (30%)	1 (25%)	
Combination	5 (8%)		
**Study methodology**			
Retrospective	10 (15%)	1 (25%)	1 (50%)
Cross-sectional	29 (43%)	3 (25%)	1 (50%)
Prospective	28 (42%)		
**Health outcomes**			
Neonatal	5 (8%)	1 (25%)	
Respiratory infections	15 (22%)		1 (50%)
Atopic conditions	26 (39%)		1 (50%)
Neurological/mental	17 (25%)	1 (25%)	
Diabetes	4 (6%)		
Combination		2 (50%)	

### Air pollution

#### Neonatal outcomes

All included studies on neonatal outcomes assess the effect of PM_2.5_ (PM less than 2.5 micrometers indiameter) or PM_10_ (PM less than 10 micrometers in diameter) exposure in mothers or the neonatal birth address. Although there has been a decreasing trend in neonatal disease burden related to PM_2.5_ in high-income regions over the past three decades, PM_2.5_ remains a notable environmental hazard for newborns [14]. Exposure to increased levels of PM_2.5_ or PM_10_ can lead to an increased risk of low birth weight or a reduction in head circumference [15–17], whereas increased levels of PM_2.5_ are also associated with decreased lung function in newborns, as measured by functional residual capacity and tidal volume [18].

#### Respiratory infections

Several studies demonstrate that short-term to medium-term exposure to pollutants such as PM_2.5_, PM_10_, NO_2_, SO_2_, and O_3_ is linked to increased hospital and emergency department (ED) admissions for viral pneumonia, bronchiolitis, bronchitis, and other respiratory infections, with cold temperatures and high humidity further exacerbating risks [19–25]. PM exposure is repeatedly associated with both higher admission rates and greater severity of bronchiolitis, while NO_2_ shows consistent associations with increased admissions too, but also heightened viral replication [26]. Other work shows PM pollution negatively affects nasal function and increases upper respiratory tract symptoms such as cough, sneezing, and a runny nose [27, 28]. Economic and population-level analyses emphasize the considerable burden of pollution-related hospitalizations for respiratory infections and subsequent respiratory morbidity in children, despite relatively low mortality rates, with costs and health service demand being substantial [29, 30]. Guillien et al. highlight that broader urban and lifestyle factors—such as limited access to natural spaces, high traffic exposure, and temperature extremes—interact with pollution to shape respiratory outcomes [31].

#### Atopic conditions

Heterogeneous results are reported about the effect of air pollutants on atopic conditions. Multiple studies demonstrate that increased exposure to different air pollutants, especially PM_2.5_ and NO_2_, prenatally and at the birth address led to an increased risk of developing asthma [32–34], but there are also studies where no association with asthma incidence or prevalence is found [35, 36]. Generally, no effect of air pollutant exposure on eczema, rhinitis, or IgE allergic sensitization is seen [32, 35, 37], expect for one study that found a positive association between PM_10_ and allergic rhinitis prevalence [36]. The effect of air pollutants on asthma incidence may be mediated through worsening lung function trajectories, as air pollutant exposure leads to reduced lung function, with underweight and obese children disproportionally affected [38–42]. Short-term O_3_ exposure was also associated with increased Fractional Exhaled Nitric Oxide (FeNO) levels in adolescents, indicating enhanced airway inflammation [43]. Longer-term studies reveal that chronic O_3_ exposure reduces lung function and increases airway inflammation and respiratory symptoms [44, 45]. Finally, air pollutants may have an effect on asthma morbidity too. Several studies show that asthmatic children experience more asthma symptoms with increased exposure to PM_10_, NO_2_, and SO_2_ [46, 47]. These symptoms may eventually lead to more health care use with positive correlations between the level of PM_2.5_, PM_10_, carbon monoxide (CO), and NO_2_ and both ED visits and hospital admissions for asthma [48–54]. Interestingly, exposure to O_3_ is frequently found to be negatively correlated to the number of consultations for asthma exacerbations [48, 49, 51].

#### Neurological and cognitive conditions

Several studies have shown the associated risks of mental or neurological problems with high exposures to air pollution perinatally or during childhood. Childhood exposure (birth to age 10) to high levels (> 26.5 µg/m^3^) of NO_2_ increases the risk for schizophrenia, irrespective of genetic factors [55, 56], and leads to increased psychopathology scores (e.g., thought disorder, externalizing and internalizing symptoms) by age 18 [57], whereas prenatal NOx exposure was positively associated with the risk of autism spectrum disorder (ASD) [58]. Ritz et al. found this association not only for NO_2_ exposure during pregnancy and infancy, but also for SO_2_ and PM_2.5_ [59]. PM_2.5_ exposure is also associated with increased risk of self-harm [60] and with lower cognitive ability at age 11 following exposure at age 3 [61]. Exposure during pregnancy and early childhood is linked to hyperactivity symptoms [62]. Exposure to air pollution (as measured by PM_2.5_ and NO_2_) in utero also negatively influences several cognitive domains [63]. Air pollution exposure to PM_2.5_, NO_2_, and PM_10_ during the first trimester of pregnancy is significantly associated with ASD and ASD severity in children [64].

#### Diabetes

Exposure to PM_10_ increases the risk of type 1 diabetes in children, with significantly higher odds among those in the highest exposure tertile [65]. Incidence was higher in regions with elevated PM_10_, and higher PM_10_ and O₃ levels were also linked to a younger age at onset. In contrast, ultrafine particles (10–30 nm) appear more relevant for adult-onset disease, with no significant associations in children [66]. Common pollutants (PM_10_, NO_2_, O_3_-AOT40) were not associated with poorer metabolic control (HbA1c). A weak inverse O_3_–HbA1c association was attributed to demographic confounding [67]. No effect of air pollutant exposure in early life on the risk and course of type 1 diabetes in children was reported [68].

### Heat stress

Several studies, mainly performed in Spain and the UK, have shown increased health issues among children during heat waves, and children seem to be among those most particularly psychologically vulnerable to the effects of heatwaves [69]. In Spain, heat-related hospitalization risk increased in infants < 1 year of age, similarly to the UK [70], where heat-related illnesses were most prevalent among children < 14 years with a threefold increase in incidence. Exposure to heatwaves in utero has also been associated with reduced birth weight and an increased risk of small-for-gestational-age (SGA) births [71].

### Wildfires

Wildfires are increasing in especially southern Europe with a possible detrimental impact on children’s health, mainly due to increasing air pollutant levels [3, 72]. Nevertheless, the additional burden posed by wildfires, on top of already elevated pollution levels, warrants a separate evaluation. In Spain, Barbosa et al. showed that an increase in NO_2_ exposure due to wildfire smoke led to an increase in bronchitis incidence and a higher symptom burden in asthmatic children, whereas an increase in PM_10_ led to a small rise in neonatal deaths as compared to baseline levels [72]. In line with this, Vicedo-Cabrera et al. found that symptoms such as itchy/watery eyes (9.4%), sneezing (8.7%), and sore throat (6.7%) were more common during the fires as opposed to a control period. Moreover, follow-up revealed that 4–6% of the children were newly diagnosed with asthma and/or rhinitis, and those with pre-existing conditions had higher risks of developing symptoms [73].

## Discussion

This scoping review synthesizes the emerging evidence on the impacts of climate change, specifically air pollution, heat exposure, and wildfires on children’s health in Europe. Our findings demonstrate that climate change is not merely a future threat but rather an urgent, multidimensional driver of pediatric morbidity, affecting developmental trajectories from the prenatal period through adolescence. Children are particularly vulnerable because of still-developing organ systems, immune system maturation, higher ventilation rates, and rapid metabolic demand [74, 75]. The evidence highlights its multidimensional impact spanning perinatal outcomes, respiratory and atopic diseases, neurological development, and mental health, all of which are compounded by socioeconomic vulnerabilities. Protecting future generations requires a coordinated action plan that prioritizes strict air-quality regulations, climate-resilient urban planning, and interventions tackling environmental injustice.

The main area of child health that has been studied includes air pollutants, which seem to have an effect not only on respiratory conditions such as asthma or infections, but also on wider organ systems including neonatal, neurological, and metabolic conditions. There seems to be a “synergy” of risks; for instance, asthmatic children exposed to both high pollen levels and high PM_10_ experience worse lung function than those exposed to either factor alone [76] and climate change may even lead to increased pollen because of a longer blooming season [77]. Remarkable is the lack of studies describing the effects of ultrafine particulate matter (PM_0.1_) [78]. Evidence for associations between heat stress and wildfires was less clear. Heat-related illnesses are prevalent particularly among children under 14, with hospitalization risks increasing significantly for infants under one year of age [69]. There is a misconception that climate warming might balance out health risks by reducing cold-related mortality. However, recent evidence suggests a seasonality reversal: the decrease in cold-attributable respiratory deaths is plateauing, while heat-attributable mortality is rising [79]. Consequently, the projected decrease in cold days will not contribute to a further reduction in respiratory deaths, requiring a re-evaluation of pediatric care during summer months. The increasing frequency of wildfires, especially in Southern Europe, illustrates the transition of climate events from local emergencies to systemic health crises.

### Strengths and limitations

This review provides a comprehensive overview of health outcomes of climate change in European children. However, limitations do exist. Firstly, heterogeneity of exposure assessment methods (e.g., land-use regression vs. monitoring stations) and outcomes restrict meta-analytic pooling. It also means that in many studies, there is a risk of bias in both exposure and outcome, leading to inconsistencies in the drawn conclusions. Moreover, assessment of the different components of climate change is also limited, as most of the included studies focus on air pollution. Nevertheless, as Europe is the fastest warming continent, heat stress and related events will be more common, and more research about its health effects is subsequently needed [80]. Additionally, there seems to be a lack of publications stemming from Eastern Europe (Fig. 2). Even though heat stress and wildfires may not be of the largest concern in this area, the disproportion in research may create an unfair representation. Furthermore, most studies are observational, given that environmental epidemiology cannot be ethically or practically studied in a randomized controlled trial. Because of this, a high level of multicollinearity remains, and a causal effect of climate change on the observed health outcome is difficult to conclude due to the lack of confounding control. Therefore, almost all studies included in this review only depict associations of climate change and children’s health. Studies assessing mitigation strategies and assessing the effectiveness of those implementations would be helpful in order to enable more robust causal inference.

### Social vulnerability

In Europe, around 24% of children live at risk of poverty or social exclusion [81]. The health impact of climate change has a larger impact on these vulnerable groups. Research showed that household income and neighborhood deprivation are linked with increased healthcare costs, which may partly be due to the impact of increased environmental exposures [82]. Children in families from a low socioeconomic position (SEP) more often live in locations that are more prone to climate change. Pollutants, such as NO_2_ and PM, are higher in larger cities and near industrial sites, where vulnerable families are overrepresented, while there is a lack of green space in these areas [83]. Health-related vulnerability has also been linked to low SEP [84] and social housing districts are often high-risk areas for heat stress [85]. In addition, Australian research showed that the health impact of climate-related disasters is larger in people living in poor-quality housing, which may be the case in Europe too [86].

### Implications for clinicians

As the evidence of the detrimental impact of climate change in Europe grows, the role of the pediatrician must evolve from treating acute symptoms solely to addressing environmental drivers and creating awareness on the impact of these drivers; see Table 2. This can be done via awareness and/or education campaigns (e.g., inviting health care providers to community centers and schools), or incorporating environmental data in clinical practice [87]. For example, initiatives in Dutch and British hospitals have led to the inclusion of individual air pollution data directly into patients’ electronic dossiers to allow clinicians to contextualize a child’s symptoms against their environmental exposure history [88, 89]. This enables health care professionals to give appropriate recommendations such as when to limit outdoor activity, which route to take to school and when to ventilate the house. Moreover, there is a need to utilize and expand diagnostic codes for environmental exposures (e.g., International Statistical Classification of Diseases and Related Health Problems codes related to air pollution) [90]. Systematically coding these factors is essential for tracking the burden of disease and validating the link between climate and health, especially given the current lack of comprehensive policy frameworks.

**Table 2:** Recommendations for (pediatric) physicians during daily practice [96]

Recommendations for (pediatric) physicians
1. Incorporate knowledge on climate change and health in curriculums of medical schools and resident education programs.
2. Counsel patients during clinic and assess their risk for their exposure to the effects of climate change. Educate patients on regional risks and protective strategies.
3. Use medical supplies appropriately, reduce pharmacological waste, use zero-emission transport travelling to work and eliminate food waste.
4. Advocate for children’s health and against climate change regionally and nationally.

### Future implications

The impact of climate change is not contained by national borders. A wildfire in Portugal, for instance, results in smoke and PM which could potentially affect air quality in neighboring countries like Spain and France [91]. Similarly, extreme temperatures do not respect geopolitical boundaries [92]. This transboundary nature necessitates a systemic, pan-European response rather than isolated local management, as climate-related disasters often have a wider reach than the immediate area of impact [93]. Nevertheless, European efforts regarding climate change mitigations are limited in pace of change [3]. In order to meet the Paris Agreement targets, the EU members have agreed to be climate neutral by 2050; however, with the current trajectory, net-zero emissions will only be met by 2100, which is driven by wind, solar, hydro and nuclear power sources [94]. Other important mitigation actions include decreasing the carbon-intensity of diets, increasing green space and maximize CO_2_ storage and sequestration [95].

## Supplementary Information

Below is the link to the electronic supplementary material.

ESM1 (DOCX 29.6 KB)

ESM2 (PDF 363 KB)

## Data Availability

No datasets were generated or analyzed during the current study.
