# Supplementary material for: Climate change and child health in Europe: a scoping review of emerging impacts
Source: Eur J Pediatr. 2026 May 5;185(6):348. doi: 10.1007/s00431-026-06966-8 (PMC13144261; doi:10.1007/s00431-026-06966-8)
Supplement: Supplementary file 1 — (DOCX 29.6 KB) [file 431_2026_6966_MOESM1_ESM.docx]

**Appendix A / Supplementary material**

**Search strategy and results**

**Database Results (11 November 2024)**

The effects of climate change on children's health in Europe. Scoping review

Berber Kapitein

Search strategies: Faridi Jamaludin, Amsterdam UMC location University of Amsterdam, Medical Library AMC, Meibergdreef 9, Amsterdam, The Netherlands

**11-11-2024:**

| Databases: | Before deduplication | After deduplication* |
| --- | --- | --- |
| PubMed, Embase (Ovid), Cochrane Library |  |  |
| Total | 4929 | 3838 |

*Lobbestael, G. (2023). DedupEndNote (Version 1.0.0) [Computer software]. https://github.com/globbestael/DedupEndNote

PUBMED

2270 results:

("Climate Change"[Mesh] OR "Air Pollution"[Mesh] OR "Environmental Exposure"[Mesh] OR "Vehicle Emissions"[Mesh] OR "Particulate Matter/adverse effects"[Mesh] OR "Particulate Matter/analysis"[Mesh] OR "Nitrogen Dioxide/adverse effects"[Mesh] OR "Nitrogen Dioxide/analysis"[Mesh] OR climate[tiab] OR air pollut*[tiab] OR wood fire*[tiab] OR heat stress*[tiab] OR flooding*[tiab] OR rainfall[tiab] OR food scarcit*[tiab] OR water scarcit*[tiab] OR crop failure*[tiab])

AND

("Child Health"[Mesh] OR "Adolescent Health"[Mesh] OR "Child"[Mesh] OR "Infant"[Mesh] OR "Adolescent"[Mesh] OR "Pediatrics"[Mesh] OR child*[tiab] OR infan*[tiab] OR newborn*[tiab] OR neonat*[tiab] OR baby[tiab] OR babies[tiab] OR pediatric*[tiab] OR paediatric*[tiab] OR adoles*[tiab] OR teen*[tiab] OR youth*[tiab] OR schoolchild*[tiab] OR preschool[tiab] OR pre-school[tiab] OR kid[tiab] OR kids[tiab] OR toddler*[tiab] OR juvenil*[tiab] OR teen*[tiab] OR pubescen*[tiab] OR puber*[tiab] OR prepubert*[tiab] OR school age*[tiab] OR schoolage*[tiab] OR elementary school[tiab] OR high school*[tiab] OR highschool*[tiab] OR kindergar*[tiab] OR boy[tiab] OR boys[tiab] OR girl*[tiab] OR minors[tiab] OR underag*[tiab] OR under ag*[tiab])

AND

("Global Health"[Mesh] OR "Global Burden of Disease"[Mesh] OR "Health Status"[Mesh] OR "Vector Borne Diseases"[Mesh] OR "Life Expectancy"[Mesh] OR "Respiratory Tract Diseases"[Mesh] OR "Respiratory Tract Infections"[Mesh] OR "Mental Health"[Mesh] OR "Stress, Psychological"[Mesh] OR "Depression"[Mesh] OR "Anxiety"[Mesh] OR "Health Status"[Mesh] OR "Socioeconomic Factors"[MeSH] OR "Social Vulnerability"[Mesh] OR health effect*[tiab] OR health impact*[tiab] OR climate change impact*[tiab] OR social stress*[tiab] OR social vulnerability[tiab] OR socioeconomic circumstance*[tiab] OR socioeconomic factor*[tiab] OR vector borne disease*[tiab] OR health disparit*[tiab] OR deprivation[tiab] OR health condition*[tiab] OR asthma[tiab])

AND

("Europe"[Mesh] OR europ*[tiab] OR netherland*[tiab] OR belgium[tiab] OR england[tiab] OR united kingdom[tiab] OR london[tiab] OR france[tiab] OR french[tiab] OR germany[tiab] OR german[tiab] OR berlin[tiab] OR scotland[tiab] OR scottish[tiab] OR wales[tiab] OR italy[tiab] OR Italian*[tiab] OR greece[tiab] OR greek[tiab] OR rome[tiab] OR spain[tiab] OR spanish[tiab] OR austria[tiab] OR scandinavia*[tiab] OR sweden[tiab] OR swedish[tiab] OR denmark[tiab] OR finland[tiab] OR iceland[tiab] OR norway[tiab] OR norwegian[tiab] OR switzerland[tiab] OR monaco[tiab] OR portugal[tiab] OR portuguese[tiab] OR andorra[tiab] OR east-europe*[tiab] OR albania[tiab] OR bulgaria*[tiab] OR croatia*[tiab] OR kosovo[tiab] OR moldavia[tiab] OR montenegro[tiab] OR belarus[tiab] OR poland[tiab] OR luxembourg[tiab] OR san marino[tiab])

AND

("2010/01/01"[Date - Publication] : "3000/11/01"[Date - Publication])

NOT

("Case Reports" [Publication Type] OR "Clinical Conference" [Publication Type] OR "Congresses as Topic"[Mesh] OR "Congress" [Publication Type] OR "Consensus Development Conference, NIH" [Publication Type] OR "Letter" [Publication Type] OR "Editorial" [Publication Type] OR case report*[ti] OR letter[ti] OR editorial[ti] OR congress*[ti] OR conference*[ti] OR "Oceania"[Mesh] OR "Oceans and Seas"[Mesh] OR "Islands"[Mesh] OR "Africa"[Mesh] OR "Americas"[Mesh] OR "Asia"[Mesh] OR saudi arabia[ti])

EMBASE (OVID):

Database(s): Embase Classic+Embase 1947 to 2024 November 08
Search Strategy:

| **#** | **Searches** | **Results** |
| --- | --- | --- |
| 1 | exp climate change/ or exp air pollution/ or exp air pollutant/ or exp environmental exposure/ or exhaust gas/ or exp particulate matter/ or nitrogen dioxide/ | 434612 |
| 2 | (climate or air pollut* or wood fire* or heat stress* or flooding* or rainfall or food scarcit* or water scarcit* or crop failure*).ti,ab,kf. | 258636 |
| 3 | 1 or 2 | 571924 |
| 4 | child/ or exp infant/ or preschool child/ or school child/ or toddler/ or adolescent/ or juvenile/ or boy/ or girl/ or exp pediatrics/ or (child* or infan* or newborn* or neonat* or baby or babies or pediatric* or paediatric* or adoles* or teen* or youth* or schoolchild* or preschool or pre-school or kid or kids or toddler* or juvenil* or pubescen* or puber* or prepubert* or school age* or schoolage* or elementary school or high school* or highschool* or kindergar* or boy or boys or girl* or minors or underag* or under ag*).ti,ab,kf. | 5909763 |
| 5 | global health/ or global disease burden/ or exp health status/ or exp vector borne disease/ or exp life expectancy/ or exp respiratory tract disease/ or exp respiratory tract infection/ or exp mental health/ or exp mental stress/ or exp mental disease/ or anxiety/ or exp socioeconomics/ or socioeconomic vulnerability/ or social vulnerability/ | 8492600 |
| 6 | (health effect* or health impact* or climate change impact* or social stress* or social vulnerability or socioeconomic circumstance* or socioeconomic factor* or vector borne disease* or health disparit* or deprivation or health condition* or asthma).ti,ab,kf. | 586355 |
| 7 | 5 or 6 | 8685792 |
| 8 | exp Europe/ or (europ* or netherland* or belgium or england or united kingdom or london or france or french or germany or german or berlin or scotland or scottish or wales or italy or Italian* or greece or greek or rome or spain or spanish or austria or scandinavia* or sweden or swedish or denmark or finland or iceland or norway or norwegian or switzerland or monaco or portugal or portuguese or andorra or east-europe* or albania or bulgaria* or croatia* or kosovo or moldavia or montenegro or belarus or poland or luxembourg or san marino).ti,ab,kf. | 3131829 |
| 9 | 3 and 4 and 7 and 8 | 5830 |
| 10 | limit 9 to yr="2010 -Current" | 3590 |
| 11 | limit 10 to conference abstracts | 662 |
| 12 | 10 not 11 | 2928 |
| 13 | case report/ or exp conference paper/ or editorial/ or erratum/ or letter/ or note/ or exp Africa/ or western hemisphere/ or exp north america/ or exp "south and central america"/ or exp Asia/ or exp Pacific islands/ or exp Middle East/ or western hemisphere/ or exp north america/ or exp "south and central america"/ or (case report* or letter or editorial or congress* or conference* or saudi arabia).ti. | 12451476 |
| 14 | 12 not 13 | 2467 |

[Cochrane Database of Systematic Reviews](https://www.cochranelibrary.com/)

Issue 11 of 12, November 2024

[Cochrane Central Register of Controlled Trials](https://www.cochranelibrary.com/)

Issue 10 of 12, October 2024

ID Search Hits

#1 (climate or air pollut* or wood fire* or heat stress* or flooding* or rainfall or food scarcit* or water scarcit* or crop failure*):ti,ab,kw 5407

#2 (child* or infan* or newborn* or neonat* or baby or babies or pediatric* or paediatric* or adoles* or teen* or youth* or schoolchild* or preschool or pre-school or kid or kids or toddler* or juvenil* or pubescen* or puber* or prepubert* or school age* or schoolage* or elementary school or high school* or highschool* or kindergar* or boy or boys or girl* or minors or underag* or under ag*):ti,ab,kw 485021

#3 (global health OR health status or vector borne disease* or life expectancy OR mental health OR stress OR depression OR anxiety OR health effect* or health impact* or climate change impact* or social stress* or social vulnerability or socioeconomic circumstance* or socioeconomic factor* or vector borne disease* or health disparit* or deprivation or health condition* or asthma or respiratory tract disease*):ti,ab,kw 514009

#4 (europ* or netherland* or belgium or england or united kingdom or london or france or french or germany or german or berlin or scotland or scottish or wales or italy or Italian* or greece or greek or rome or spain or spanish or austria or scandinavia* or sweden or swedish or denmark or finland or iceland or norway or norwegian or switzerland or monaco or portugal or portuguese or andorra or east-europe* or albania or bulgaria* or croatia* or kosovo or moldavia or montenegro or belarus or poland or luxembourg or san marino):ti,ab,kw 170520

#5 #1 and #2 and #3 and #4 in Cochrane Reviews, Trials 192
