# Supplementary material for: Climate change and child health in Europe: a scoping review of emerging impacts
Source: Eur J Pediatr. 2026 May 5;185(6):348. doi: 10.1007/s00431-026-06966-8 (PMC13144261; doi:10.1007/s00431-026-06966-8)
Supplement: Supplementary file 2 — (PDF 363 KB) [file 431_2026_6966_MOESM2_ESM.pdf]

Author, year	Country	Study design	Number of patients	Study population	Study variables	Outcome variables
AIR POLLUTION						
Neonatal outcomes						
Arroyo et al., 2016[1]	Spain	Cross-sectional	298,705 births	Neonates	Mean daily $PM_{2.5}$, NO_2 and O_3 (µg/m3), maximum and minimum daily temperatures (°C)	LBW (<2500 g), premature birth (<37 weeks of gestation), and late fetal death (<24 h of life)
Pedersen et al. 2016[2]	Several European cohorts	Cross-sectional	34,923 singleton births in 1994–2008	Neonates	Annual average concentrations of elemental constituents of $PM_{2.5}$ and PM_{10} at maternal home addresses during pregnancy	Birth weight
Rasking et al. 2024[3]	Belgium	Prospective cohort	1484	Mother–neonate pairs	Black carbon and $PM_{2.5}$ levels of the mother's residential address(es)	Cord blood cystatin C as measurement of neonatal kidney function
Ren et al. 2023[4]	Global	Retrospective cohort	Global data per 100.000 individuals	Neonates	$PM_{2.5}$	Age-standardized mortality rate; neonatal preterm birth; neonatal encephalopathy due to birth asphyxia and trauma; neonatal sepsis and other neonatal infections; HDNJ: hemolytic disease and other neonatal jaundice
Rittner et al. 2020[5]	Sweden	Retrospective analysis	1,247,993 individuals	All individuals of Scania > 5 years of age	$PM_{2.5}$ concentration of a 100 × 100 m cell in which a patient's centroid of their residence was located	Birthweight and premature deaths
Respiratory infections						

Álvaro-Meca et al., 2022[6]	Spain	Bidirectional case-crossover study	30.445	Children <2 years	Temperature, relative humidity, NO_2, SO_2, O_3 and PM_{10}-levels	Hospital admissions for acute viral LRTIs
Bono et al., 2016[7]	Italy	Prospective cohort	20512 (excl 1281 asthma)	Children aged 0-18 years	Temperature, relative humidity, cumulative daily precipitations, NO_2, $PM_{2.5}$, O_3, and aeroallergens	ED-admissions for respiratory diseases
Carlsen et al., 2016[8]	Sweden	Prospective cohort	95	Children aged 11-12 years	$PM_{2.5}$, $PM_{2.5–10}$, O_3, NO_2, NO_x, NO	Airway inflammation ($FENO_{50}$)
Carugno et al., 2018[9]	Italy	Retrospective time-series study	2814	Children aged 0-18 years	PM_{10}	Hospitalization due to RSV-bronchiolitis
Pablo-Romero et al., 2015[10]	Spain	Retrospective cohort	2043 (excluding 87 asthma patients)	Children < 14 years	$PM_{2.5}$, temperature, relative humidity	Hospital admissions for respiratory diseases
Dimakopoulou et al., 2020[11]	Greece	Panel study	186	Children aged 10-11 years	O_3, PM_{10}, time spent outdoors (obtained through time activity diaries)	Long-term effects on lung function (spirometry) and FeNO levels.
Gallo et al., 2023[12]	Italy	Retrospective database	2215	Children < 1 years	$Pm_{2.5}$, PM_{10}, NO_2	Bronchiolitis severity
Guillien et al., 2024[13]	UK, France, Spain, Lithuania, Norway and Greece	Prospective cohort	1033	Children aged 6-12 years	$Pm_{2.5-10}$, NO_2, greenness, building density, VOCS	Lung function, ISAAC questionnaires, rhinitis
Kanellopoulos et al., 2021[14]	Greece	Retrospective analysis of prospectively collected data	4017 (excl 208 asthma)	Children aged 0-18 years	$PM_{2.5}$	ED visits for respiratory related issues
Karakatsani et al., 2017[15]	Greece	Panel study	188	Children aged 10-11 years	O_3 exposure	Respiratory symptoms, Lung Function
Milani et al., 2022[16]	Italy	Prospective cohort	110	Children < 1 year	The daily PM_{10} and $PM_{2.5}$ exposure in the 29 preceding days of measurement	Bronchiolitis severity score

Ratajczak et al., 2021[17]	Poland	Prospective cohort	?	Children aged 3–12 years	Daily changes in PM_{10} and $PM_{2.5}$	The number of children reporting URTS and their severity.
Shabani Isenaj et al., 2022[18]	Kosovo	Retrospective data base	1838 hospital admission records and 7372 ambulatory visits	Children aged 0–18 years	$PM_{2.5}$	Ambulatory and ER visits due to respiratory symptoms
Van Brusselen et al., 2024[19]	Belgium	Prospective cohort	118 cases and 79 controls	Children < 2 years	Predicted values of $PM_{2.5}$, PM_{10}, BC and NO_2 exposure, at the home address, and daycare address of the participant 1 to 5 days prior to hospitalization	Hospitalization for bronchiolitis
Atopic conditions						
Altzibar et al., 2014[20]	Spain	Observational study	28.477	Adults, but also children <15 years	Weekly average exposure to CO, PM_{10}, $PM_{2.5}$, NO_2 and O_3, temperature, relative humidity	ED visits and hospitalizations for asthma exacerbations
Amazouz et al., 2021[21]	France	Prospective birth cohort study	1063	Children at age 8 years old	Daily total pollen concentration, daily allergenic risk indices for specific pollen taxa, daily concentrations of PM_{10}, NO_2 and O_3 up to 4 days before the day of measurement	Lung function, FeNO
Ashworth et al., 2021[22]	England	Observational study	Not given	Adults, but also children <18 years	Daily PM_{10}, $PM_{2.5}$, NO_2 and O_3	Respiratory consultations for asthma and URTIs
Bouazza et al., 2018[23]	France	Observational study	47.107	Children <18 years	Daily mean exposure to concentration of PM_{10}, $PM_{2.5}$, NO_2 and O_3	ED visits for asthma exacerbations
Gehring et al., 2015[24]	Germany, Sweden, The Netherlands	Prospective birth cohort study	14.126	Children up to age 14-16 years	Annual average concentrations of PM_{10}, $PM_{2.5}$, NO_2, PM_{coarse}, and $PM_{2.5}$ absorbance at home address level	Incidence of asthma and rhino conjunctivitis

Gehring et al., 2015[25]	Netherlands	Prospective cohort	3702	Children aged 0-12 years	$PM_{2.5}$ and $_{10}$ and specific compounds such as copper, iron, potassium.	Asthma and allergy
Kouis et al., 2024[26]	Cyprus, Greece	Randomized controlled trial	182	Children aged 6-11 years with asthma	Interventions targeting exposure to outside concentrations of PM_{10} (one control group, one outdoor intervention group and one combined intervention group)	Asthma symptom control (c-ACT), lung function, FeNO
Lepeule et al. 2023[27]	France	Prospective longitudinal cohort	391	Infants 2 months of age	NO_2, $PM_{2.5}$ measured by wearables by mothers	Lung function in newborns
Mejias et al., 2019[28]	Spain	Observational retrospective study	2609	Children <16 years	Daily pollution data of NO_2, PM_{10} and O_3 at a monitoring station close to the hospital	ED visits for an asthma exacerbation
Mazenq et al., 2017[29]	France	Nested case-control study (1:15)	68.897	Children between 3-18 years of age	PM_{10}, $PM_{2.5}$ and meteorological conditions linked to postal code	Risk of asthma emergency visits
Melen et al., 2020[30]	Germany, Sweden, The Netherlands	Prospective birth cohort study	6.163	Children up to age 16 years	Individual residential outdoor levels of PM_{10}, $PM_{2.5}$, $PM_{2.5}$ absorbance, NO_2 and NO_x	Serum IgE sensitization to allergen extracts
Milanzi et al., 2018[31]	The Netherlands	Prospective birth cohort study	1.636	Children up to age 16 years	Annual average concentrations of PM_{10}, $PM_{2.5}$, NO_2, PM_{coarse}, and $PM_{2.5}$ absorbance at home address level	Lung function
Patlán-Hernández et al., 2024[32]	France	Prospective mother-child cohort	2.440	Children at 6- and 12-years of age	Exposure during pregnancy to $PM_{2.5}$ and NO_2 at the residential address	Incidence of asthma, rhinitis, eczema
Paunescu et al., 2019[33]	France, Spain	Prospective birth cohort	305	10-year-old children with and without persistent wheezing and/ or asthma attacks	Personal 24-hour measurements of exposure to BC and UFP	Lung function, FeNO
Pedersen et al., 2023[34]	Denmark	Nationwide prospective birth cohort	1.060.154	Children aged 0-19 years	Monthly mean concentrations of O_3, NO_2, NO_3^-, NO_x, PM_{10}, $PM_{2.5}$, SO_2, SO_4^-, NH_3, NH_4^+,	Incidence of asthma

					secondary inorganic aerosols (sum of NO_3^-, SO_4^- and NH_4^+), elemental carbon, organic carbon, sea salt	
Puklova et al., 2019[35]	Czech Republic	Cross-sectional study	7.239	Children at 5, 9, 13 and 17 years	Exposure to PM_{10} and NO_2 at the residential address	Prevalence of respiratory allergic diseases
Ranzi et al., 2014[36]	Italy	Prospective cohort study	69	Asthmatic children aged 6-7 years	Individual exposure at address level of PM_{10}	Respiratory symptoms
Spyratos et al., 2015[37]	Greece	Prospective cohort study	1425	Children aged 10-12 years	Concentrations of PM_{10} as well as SO_2	Asthma, Rhinitis, Allergy
Usemann et al., 2019[38]	Switzerland	Prospective birth cohort	232	Children at 6 years	Individual level PM_{10}, O_3 and NO_2 during pregnancy until 6 years	Lung function
Velická et al., 2015[39]	Czech Republic	Prospective cohort study	147	Children aged 6-18 years old with mild to moderate persistent asthma	Daily exposure based on time spent at home and school NO_2, PM_{10} and SO_2	Respiratory symptoms
Veremchuk et al., 2015[40]	Russia	Prospective cohort study	Not given	Children and adolescents	Aerosol suspension of PM, NO_2, SO_2, CO, NH_3 and CH_2O in snow and air, climatic factors	ED visits for asthma exacerbations
Wang et al., 2024[41]	England	Time-stratified case-crossover study	111.766	Children between 0-14 years	Daily NO_2 levels at the residential address with land use data and chemical transport estimates	Emergency hospital admissions for asthma
Yu et al., 2023[42]	Sweden	Prospective birth cohort	1.509	Children and adolescents between 8, 16 and 24 years old	Exposure to $PM_{2.5}$, PM_{10}, BC and NO_x at residential addresses	Lung function
Yu et al., 2024[43]	Sweden	Prospective birth cohort	2.371	Children and adolescents between 8-24 years old	Change in individual-level exposure to $PM_{2.5}$, PM_{10}, BC and NO_x at residential addresses	Incidence of asthma
Zhao et al., 2019[44]	Germany	Prospective birth cohort	2.921	10-year-old and 15-year-old children	Maximum 8 our averages of O_3 and daily average	FeNO

					concentrations of NO_2 and PM_{10} at monitoring stations	
Zhao et al., 2021[45]	Germany	Prospective birth cohort	915	Children up until 15 years of age	Annual average concentrations of NO_2, $PM_{2.5}$, PM_{10}, PM_{coarse} and $PM_{absorbance}$ in the first year of life	Lung function
Zhao et al., 2021[46]	Germany	Prospective birth cohort	2.224	15-year-old children	Air pollutant exposure of $PM_{2.5}$, PM_{10}, $PM_{2.5}$ absorbance, PM_{coarse} and NO_2 at 6, 10 and 15 years	Lung function
Neurological and cognitive conditions						
Antonsen et al. (2020)[47]	Denmark	Cohort	2189	Children up until 10 years	Annual average concentrations of NO_2, $PM_{2.5}$, PM_{10}	Schizophrenia
Baranyi et al. (2023)[48]	Scotland	Cohort	2,734	Age 11-86	$PM_{2.5}$ exposure	Cognitive ability and mortality
Binter et al. (2022)[49]	Netherlands	Cohort	3515	Children between 9-12 years	Outdoor air pollutants (NO_2, $PM_{2.5}$, and $PM_{2.5}$ absorbance)	Brain development; white matter, brain volume
Chen et al. (2024)[50]	Spain	Cohort	1416	Children up until 7 years	$PM_{2.5}$	ADHD
Horsdal et al. (2019)[51]	Denmark	Case-cohort	3531	Children up until 10 years	NO_2	Schizophrenia
Kusters et al. (2022)[52]	Netherlands	Cohort	5170	Children up until adolescence	Cu, OC, Fe, OP of PM, and PM_{coarse}	Cognitive function and behavioral problems
Kusters et al. (2024)[53]	Netherlands	Cohort	4108	Children 10-14 years old	$PM_{2.5}$, PM_{10}, PM_{coarse}, NO_x, OPDTT	White matter microstructure development
Lertxundi et al. 2019[54]	Spain	Prospective longitudinal cohort	1119	Mother-child pairs Children 4-6 years	NO_2, $PM_{2.5}$ measured at postal code during pregnancy	Infants' neuropsychological development
Lubczyńnska et al. (2020)[55]	Netherlands	Cohort	2954	Children 9-12 years old	Prenatal exposure to $PM_{2.5}$, elemental Si, NO_x, elemental Zn and OP of PM	White matter microstructure development

Lubczyńska, et al. (2020)[56]	Netherlands	Cohort	3133	Children 9-12 years old	NO_x, $PM_{2.5}$, PM_{10} and PM_{coarse}	Brain morphology
Mok et al. (2021)[57]	Denmark	National cohort	32,984	Children until 10 years	$PM_{2.5}$ and NO_2	Mental illness and self-harm
Oudin et al. (2016)[58]	Sweden	Longitudinal cohort	552,221	Children until 18 years	NO_2, PM_{10}, and $PM_{2.5}$	Psychiatric disorders
Oudin et al. (2019)[59]	Sweden	Longitudinal cohort	48,571	Children until 18 years	NO_x	Autism and ADHD
Reuben et al. (2021)[60]	England and Wales	Longitudinal cohort	2039	Children 10-18 years	$PM_{2.5}$ and NO_x	Psychopathology
Ritz et al. (2018)[61]	Denmark	Cohort	15387	9 months before to 9 months after pregnancy	NO_2, SO_2, $PM_{2.5}$ and PM_{10}	Autism spectrum disorder
Roberts et al. (2019)[62]	UK	cohort of twins from the E-Risk Longitudinal Study	284	Children until 18 years	NO, NO_2, NO_x, O_3, O_x, PM_{10}, $PM_{2.5}$, and PM_{coarse}	Mental health problems
Santos et al. 2023[63]	Portugal	Retrospective cohort	217	Children <17 years	O_3, $PM_{2.5}$, PM_{10}, NO_2, SO_2	Autism severity
Diabetes						
Badpa et al., 2022[64]	Germany	Cohort	included 85,251 and 272 diagnosed	Children until 6 years	Daily averages for $PM_{2.5}$, NO_2, O_3, temperature	Type 1 diabetes
Di Ciaula (2015)[65]	Italy	Observational study with time-trend analysis	1501	Children until 14 years	Average PM_{10}, O_3, NO_x and CO levels	Type 1 diabetes
Hrženjak et al., 2020[66]	Slovenia	5-year time-trend study	population between approximately 282,741 and 288,250 people over the period 2013–2017	Children 0-10 years and elderly 60+ years	Daily UFP, $PM_{2.5}$ and PM_{10} concentrations	Primary Health Care Consultations for Diabetes
Tamayo et al., 2016[67]	Germany	Retrospective cohort	771	Children 11-21 years	PM_{10}, NO_2, and O_3-AOT40	Diabetes type 1
HEAT STRESS						

Achebak et al. (2024)[68]	Spain	Cross sectional study	Over 11 million ED emissions	All ages, with a focus on infants <1 years of age	Weather (i.e., temperature and relative humidity), $PM_{2.5}$, PM_{10}, NO_2, and O_3	Emergency department visits
Christodoulou et al. (2024)[69]	France	Time-series observational study	1,198,953	6% children aged 0-15 years	Weather conditions	Conduct disorder, anxiety, mood disorder
Rancière et al. (2024)[70]	France	Cohort study	3359 mothers	Birth	Heat wave experienced by the mothers	Number of neonates with small for gestational age weight
Smith et al. (2016)[71]	UK	Retrospective observational study	1166 during heatwave year	Children aged 0-4 years and 5-14 years	Heat wave period	Emergency department visits for heat illness
**WILDFIRES**						
Barbosa et al. (2022)[72]	Spain	Retrospective observational study	320 cases in 2016 and 648 in 2017	Children until 18 years	Wildfires	Bronchitis and mortality
Vicedo-Cabrera et al. (2016)[73]	Spain	Cohort study	460	Children until 18 years	Two wildfires	Itchy/watery eyes, sneezing, sore throat, asthma and/or rhinitis

Table E1: Outcomes of the included studies of this review

BC = black carbon; CH2O = formaldehyde; CO = carbon monoxide; Cu = copper; ED = emergency department; Fe = iron; FeNO = fractional exhaled nitric oxide; LBW = low birth weight; LTRIs = lower respiratory tract infections; NO = nitrogen oxide; NO2 = nitric dioxide; NO3- = nitrate; NOx = both nitrogen oxide and nitrogen dioxide; OP = oxidative potential; OC = organic carbon; Ox = ozone and nitrogen dioxide; O3 = ozone; O3-AOT40 = accumulated ozone; PAH = polycyclic aromatic hydrocarbons; PMcoarse = particulate matter with a diameter between 2.5-10 PM2.5 = particulate matter with a diameter ≤2.5 micrometers; PM10 = particulate matter with a diameter ≤10 micrometers; Si = silicon; SNP = single nucleotide polymorphisms; SO2 = sulphur dioxide; UFP = ultrafine particles; U.K. = United Kingdom; URTIs = upper respiratory tract infections; Zn = zinc.

1. Arroyo V, Diaz J, Carmona R, Ortiz C, Linares C. Impact of air pollution and temperature on adverse birth outcomes: Madrid, 2001-2009. Environ Pollut. 2016;218:1154–61.
2. Pedersen M, Gehring U, Beelen R, Wang M, Giorgis-Allemand L, Andersen AM, et al. Elemental Constituents of Particulate Matter and Newborn's Size in Eight European Cohorts. Environ Health Perspect. 2016;124(1):141–50.
3. Rasking L, Van Pee T, Vangeneugden M, Renaers E, Wang C, Penders J, et al. Newborn glomerular function and gestational particulate air pollution. eBioMedicine. 2024;107:105253.
4. Ren B, He Q, Ma J, Zhang G. A preliminary analysis of global neonatal disorders burden attributable to PM(2.5) from 1990 to 2019. Sci Total Environ. 2023;870:161608.
5. Rittner R, Flanagan E, Oudin A, Malmqvist E. Health Impacts from Ambient Particle Exposure in Southern Sweden. Int J Environ Res Public Health. 2020;17(14):1–12.
6. Álvaro-Meca A, Goez MDC, Resino R, Matías V, Sepúlveda-Crespo D, Martínez I, et al. Environmental factors linked to hospital admissions in young children due to acute viral lower respiratory infections: A bidirectional case-crossover study. Environ Res. 2022;212(Pt B):113319.
7. Bono R, Romanazzi V, Bellisario V, Tassinari R, Trucco G, Urbino A, et al. Air pollution, aeroallergens and admissions to pediatric emergency room for respiratory reasons in Turin, northwestern Italy. BMC Public Health. 2016;16:722.
8. Carlsen HK, Boman P, Bjor B, Olin AC, Forsberg B. Coarse fraction particle matter and exhaled nitric oxide in non-asthmatic children. International Journal of Environmental Research and Public Health. 2016;13(6).
9. Carugno M, Dentali F, Mathieu G, Fontanella A, Mariani J, Bordini L, et al. PM10 exposure is associated with increased hospitalizations for respiratory syncytial virus bronchiolitis among infants in Lombardy, Italy. Environ Res. 2018;166:452–7.
10. de PP-RM, Román R, Limón JM, Praena-Crespo M. Effects of fine particles on children's hospital admissions for respiratory health in Seville, Spain. J Air Waste Manag Assoc. 2015;65(4):436–44.
11. Dimakopoulou K, Douros J, Samoli E, Karakatsani A, Rodopoulou S, Papakosta D, et al. Long-term exposure to ozone and children's respiratory health: Results from the RESPOZE study. Environ Res. 2020;182:109002.
12. Gallo E, Bressan S, Baraldo S, Bottigliengo D, Geremia S, Acar AS, et al. Increased risk of emergency department presentations for bronchiolitis in infants exposed to air pollution. Risk Anal. 2023;43(6):1137–44.
13. Guillien A, Slama R, Andrusaityte S, Casas M, Chatzi L, de Castro M, et al. Associations between combined urban and lifestyle factors and respiratory health in European children. Environ Res. 2024;242:117774.
14. Kanellopoulos N, Pantazopoulos I, Mermiri M, Mavrovounis G, Kalantzis G, Saharidis G, et al. Effect of PM(2.5) Levels on Respiratory Pediatric ED Visits in a Semi-Urban Greek Peninsula. Int J Environ Res Public Health. 2021;18(12):6384.
15. Karakatsani A, Samoli E, Rodopoulou S, Dimakopoulou K, Papakosta D, Spyratos D, et al. Weekly Personal Ozone Exposure and Respiratory Health in a Panel of Greek Schoolchildren. Environ Health Perspect. 2017;125(7):077016.
16. Milani GP, Cafora M, Favero C, Luganini A, Carugno M, Lenzi E, et al. PM(2) (.5,) PM(10) and bronchiolitis severity: A cohort study. Pediatr Allergy Immunol. 2022;33(10):e13853.
17. Ratajczak A, Badyda A, Czechowski PO, Czarnecki A, Dubrawski M, Feleszko W. Air pollution increases the incidence of upper respiratory tract symptoms among polish children. Journal of Clinical Medicine. 2021;10(10):2150.

18. Shabani Isenaj Z, Berisha M, Gjorgjev D, Dimovska M, Moshammer H, Ukëhaxhaj A. Air Pollution in Kosovo: Short Term Effects on Hospital Visits of Children Due to Respiratory Health Diagnoses. Int J Environ Res Public Health. 2022;19(16):10141.
19. Van Brusselen D, De Troeyer K, van Vliet MP, Avonts D, Nemery B, Liesenborghs L, et al. Air pollution and bronchiolitis: a case-control study in Antwerp, Belgium. Eur J Pediatr. 2024;183(5):2431–42.
20. Altzibar JM, Tamayo-Uria I, De Castro V, Aginagalde X, Albizu MV, Lertxundi A, et al. Epidemiology of asthma exacerbations and their relation with environmental factors in the Basque Country. Clin Exp Allergy. 2015;45(6):1099–108.
21. Amazouz H, Bougas N, Thibaudon M, Lezmi G, Beydon N, Bourgoin-Heck M, et al. Association between lung function of school age children and short-term exposure to air pollution and pollen: the PARIS cohort. Thorax. 2021;76(9):887–94.
22. Ashworth M, Analitis A, Whitney D, Samoli E, Zafeiratou S, Atkinson R, et al. Spatio-temporal associations of air pollutant concentrations, GP respiratory consultations and respiratory inhaler prescriptions: a 5-year study of primary care in the borough of Lambeth, South London. Environ Health. 2021;20(1):54.
23. Bouazza N, Foissac F, Urien S, Guedj R, Carbajal R, Tréluyer JM, et al. Fine particulate pollution and asthma exacerbations. Arch Dis Child. 2018;103(9):828–31.
24. Gehring U, Wijga AH, Hoek G, Bellander T, Berdel D, Brüske I, et al. Exposure to air pollution and development of asthma and rhinoconjunctivitis throughout childhood and adolescence: a population-based birth cohort study. Lancet Respir Med. 2015;3(12):933–42.
25. Gehring U, Beelen R, Eeftens M, Hoek G, de Hoogh K, de Jongste JC, et al. Particulate matter composition and respiratory health: the PIAMA Birth Cohort study. Epidemiology. 2015;26(3):300–9.
26. Kouis P, Galanakis E, Michaelidou E, Kinni P, Michanikou A, Pitsios C, et al. Improved childhood asthma control after exposure reduction interventions for desert dust and anthropogenic air pollution: the MEDEA randomised controlled trial. Thorax. 2024;79(6):495–507.
27. Lepeule J, Pin I, Boudier A, Quentin J, Lyon-Caen S, Supernant K, et al. Pre-natal exposure to NO2 and PM2.5 and newborn lung function: An approach based on repeated personal exposure measurements. Environmental Research. 2023;226:115656.
28. Marques Mejías MA, Tomás Pérez M, Hernández I, López I, Quirce S. Asthma Exacerbations in the Pediatric Emergency Department at a Tertiary Hospital: Association With Environmental Factors. J Investig Allergol Clin Immunol. 2019;29(5):365–70.
29. Mazenq J, Dubus JC, Gaudart J, Charpin D, Nougairede A, Viudes G, et al. Air pollution and children's asthma-related emergency hospital visits in southeastern France. Eur J Pediatr. 2017;176(6):705–11.
30. Melén E, Standl M, Gehring U, Altug H, Antó JM, Berdel D, et al. Air pollution and IgE sensitization in 4 European birth cohorts-the MeDALL project. J Allergy Clin Immunol. 2021;147(2):713–22.
31. Milanzi EB, Koppelman GH, Smit HA, Wijga AH, Oldenwening M, Vonk JM, et al. Air pollution exposure and lung function until age 16 years: the PIAMA birth cohort study. Eur Respir J. 2018;52(3).
32. Patlán-Hernández AR, Savouré M, Audureau E, Monfort C, de Castro M, Epaud R, et al. Associations of exposure to outdoor PM(2.5) and NO(2) during pregnancy with childhood asthma, rhinitis, and eczema in a predominantly rural French mother-child cohort. Environ Pollut. 2024;363(Pt 2):125206.
33. Paunescu AC, Casas M, Ferrero A, Pañella P, Bougas N, Beydon N, et al. Associations of black carbon with lung function and airway inflammation in schoolchildren. Environ Int. 2019;131:104984.

34. Pedersen M, Liu S, Zhang J, Andersen ZJ, Brandt J, Budtz-Jorgensen E, et al. Early-Life Exposure to Ambient Air Pollution from Multiple Sources and Asthma Incidence in Children: A Nationwide Birth Cohort Study from Denmark. Environmental Health Perspectives. 2023;131(5):057003.
35. Puklová V, Žejglicová K, Kratěnová J, Brabec M, Malý M. Childhood respiratory allergies and symptoms in highly polluted area of Central Europe. Int J Environ Health Res. 2019;29(1):82–93.
36. Ranzi A, Ancona C, Angelini P, Badaloni C, Cernigliaro A, Chiusolo M, et al. [Health impact assessment of policies for municipal solid waste management: findings of the SESPIR Project]. Epidemiol Prev. 2014;38(5):313–22.
37. Spyratos D, Sioutas C, Tsiotsios A, Haidich AB, Chloros D, Triantafyllou G, et al. Effects of particulate air pollution on nasal and lung function development among Greek children: a 19-year cohort study. Int J Environ Health Res. 2015;25(5):480–9.
38. Usemann J, Decrue F, Korten I, Proietti E, Gorlanova O, Vienneau D, et al. Exposure to moderate air pollution and associations with lung function at school-age: A birth cohort study. Environ Int. 2019;126:682–9.
39. Velická H, Puklová V, Keder J, Brabec M, Malý M, Bobák M, et al. Asthma Exacerbations and Symptom Variability in Children Due to Short-term Ambient Air Pollution Changes in Ostrava, Czech Republic. Cent Eur J Public Health. 2015;23(4):292–8.
40. Veremchuk LV, Cherpack NA, Gvozdenko TA, Volkova MV. METHODOLOGY FOR THE ASSESSMENT OF THE IMPACT OF THE ATMOSPHERIC AIR POLLUTION ON THE FORMATION OF THE LEVELS OF OVERALL MORBIDITY RATE OF BRONCHIAL ASTHMA. Gigiena i sanitariia. 2015;94(3):119–22.
41. Wang W, Gulliver J, Beevers S, Freni Sterrantino A, Davies B, Atkinson RW, et al. Short-Term Nitrogen Dioxide Exposure and Emergency Hospital Admissions for Asthma in Children: A Case-Crossover Analysis in England. J Asthma Allergy. 2024;17:349–59.
42. Yu Z, Merid SK, Bellander T, Bergström A, Eneroth K, Georgelis A, et al. Associations of improved air quality with lung function growth from childhood to adulthood: the BAMSE study. Eur Respir J. 2023;61(5):2201783.
43. Yu Z, Kebede Merid S, Bellander T, Bergström A, Eneroth K, Merritt AS, et al. Improved Air Quality and Asthma Incidence from School Age to Young Adulthood: A Population-based Prospective Cohort Study. Ann Am Thorac Soc. 2024;21(10):1432–40.
44. Zhao T, Markevych I, Standl M, Schikowski T, Berdel D, Koletzko S, et al. Short-term exposure to ambient ozone and inflammatory biomarkers in cross-sectional studies of children and adolescents: Results of the GINIplus and LISA birth cohorts. Environmental Pollution. 2019;255:113264.
45. Zhao Q, Kress S, Markevych I, Berdel D, von Berg A, Gappa M, et al. Air pollution during infancy and lung function development into adolescence: The GINIplus/LISA birth cohorts study. Environ Int. 2021;146:106195.
46. Zhao Q, Kress S, Markevych I, Berdel D, von Berg A, Gappa M, et al. Long-term Air Pollution Exposure Under European Union Limits and Adolescents' Lung Function: Modifying Effect of Abnormal Weight in the GINIplus and LISA Birth Cohorts. Chest. 2021;160(1):249–58.
47. Antonsen S, Mok PLH, Webb RT, Mortensen PB, McGrath JJ, Agerbo E, et al. Exposure to air pollution during childhood and risk of developing schizophrenia: a national cohort study. The Lancet Planetary Health. 2020;4(2):e64–e73.
48. Baranyi G, Williamson L, Feng Z, Tomlinson S, Vieno M, Dibben C. Early life PM2.5 exposure, childhood cognitive ability and mortality between age 11 and 86: A record-linkage life-course study from Scotland. medRxiv. 2023.
49. Binter AC, Kusters MSW, van den Dries MA, Alonso L, Lubczynska MJ, Hoek G, et al. Air pollution, white matter microstructure, and brain volumes: Periods of susceptibility from pregnancy to preadolescence. Environmental Pollution. 2022;313:120109.
50. Chen WJ, Rector-Houze AM, Guxens M, Iniguez C, Swartz MD, Symanski E, et al. Susceptible windows of prenatal and postnatal fine particulate matter exposures and attention-deficit hyperactivity disorder symptoms in early childhood. Science of the Total Environment. 2024;912:168806.

51. Horsdal HT, Agerbo E, McGrath JJ, Vilhjalmsson BJ, Antonsen S, Closter AM, et al. Association of Childhood Exposure to Nitrogen Dioxide and Polygenic Risk Score for Schizophrenia with the Risk of Developing Schizophrenia. JAMA Network Open. 2019;2(11):e1914401.
52. Kusters MSW, Essers E, Muetzel R, Ambros A, Tiemeier H, Guxens M. Air pollution exposure during pregnancy and childhood, cognitive function, and emotional and behavioral problems in adolescents. Environmental Research. 2022;214:113891.
53. Kusters MSW, Lopez-Vicente M, Muetzel RL, Binter AC, Petricola S, Tiemeier H, et al. Residential ambient air pollution exposure and the development of white matter microstructure throughout adolescence. Environmental Research. 2024;262:119828.
54. Lertxundi A, Andiarena A, Martinez MD, Ayerdi M, Murcia M, Estarlich M, et al. Prenatal exposure to PM2.5 and NO2 and sex-dependent infant cognitive and motor development. Environmental Research. 2019;174:114–21.
55. Lubczynnska MJ, Muetzel RL, Marroun HE, Basagana X, Strak M, Denault W, et al. Exposure to air pollution during pregnancy and childhood, and white matter microstructure in preadolescents. Environmental Health Perspectives. 2020;128(2):027005.
56. Lubczynska MJ, Muetzel RL, El Marroun H, Hoek G, Kooter IM, Thomson EM, et al. Air pollution exposure during pregnancy and childhood and brain morphology in preadolescents. Environmental Research. 2021;198:110446.
57. Mok PLH, Antonsen S, Agerbo E, Brandt J, Geels C, Christensen JH, et al. Exposure to ambient air pollution during childhood and subsequent risk of self-harm: A national cohort study. Preventive medicine. 2021;152:106502.
58. Oudin A, Bråbäck L, Åström DO, Strömgren M, Forsberg B. Association between neighbourhood air pollution concentrations and dispensed medication for psychiatric disorders in a large longitudinal cohort of Swedish children and adolescents. BMJ Open. 2016;6(6):e010004.
59. Oudin A, Frondelius K, Haglund N, Kallen K, Forsberg B, Gustafsson P, et al. Prenatal exposure to air pollution as a potential risk factor for autism and ADHD. Environment International. 2019;133:105149.
60. Reuben A, Arseneault L, Beddows A, Beevers SD, Moffitt TE, Ambler A, et al. Association of Air Pollution Exposure in Childhood and Adolescence With Psychopathology at the Transition to Adulthood. JAMA network open. 2021;4(4):e217508.
61. Ritz B, Liew Z, Yan Q, Cuia X, Virk J, Ketzel M, et al. Air pollution and autism in Denmark. Environmental Epidemiology. 2018;2(4):e028.
62. Roberts S, Arseneault L, Barratt B, Beevers S, Danese A, Odgers CL, et al. Exploration of NO2 and PM2.5 air pollution and mental health problems using high-resolution data in London-based children from a UK longitudinal cohort study. Psychiatry Research. 2019;272:8–17.
63. Santos JX, Sampaio P, Rasga C, Martiniano H, Faria C, Cafe C, et al. Evidence for an association of prenatal exposure to particulate matter with clinical severity of Autism Spectrum Disorder. Environmental Research. 2023;228:115795.
64. Badpa M, Wolf K, Schneider A, Winkler C, Haupt F, Peters A, et al. Association of long-term environmental exposures in pregnancy and early life with islet autoimmunity development in children in Bavaria, Germany. Environ Res. 2022;212(Pt D):113503.
65. Di Ciaula A. Type I diabetes in paediatric age in Apulia (Italy): Incidence and associations with outdoor air pollutants. Diabetes Research and Clinical Practice. 2016;111:36–43.
66. Hrzenjak VV, Kukec A, Erzen I, Stanimirovic D. Effects of ultrafine particles in ambient air on primary health care consultations for diabetes in children and elderly population in ljubljana, Slovenia: A 5-year time-trend study. International Journal of Environmental Research and Public Health. 2020;17(14):1–19.
67. Tamayo T, Rathmann W, Stahl-Pehe A, Landwehr S, Sugiri D, Krämer U, et al. No adverse effect of outdoor air pollution on HbA1c in children and young adults with type 1 diabetes. Int J Hyg Environ Health. 2016;219(4-5):349–55.

68. Achebak H, Rey G, Chen ZY, Lloyd SJ, Quijal-Zamorano M, Mendez-Turrubiates RF, et al. Heat Exposure and Cause-Specific Hospital Admissions in Spain: A Nationwide Cross-Sectional Study. Environmental Health Perspectives. 2024;132(5):R version 3.4.3.

69. Christodoulou N, Laaidi K, Fifre G, Lejoyeux M, Ambar Akaoui M, Geoffroy PA. Heatwaves and mental disorders: A study on national emergency and weather services data. European Journal of Psychiatry. 2024;38(3):100249.

70. Ranciere F, Wafo O, Perrot X, Momas I. Associations between heat wave during pregnancy and term birth weight outcomes: The PARIS birth cohort. Environment International. 2024;188:108730.

71. Smith S, Elliot AJ, Hajat S, Bone A, Bates C, Smith GE, et al. The impact of heatwaves on community morbidity and healthcare usage: A retrospective observational study using real-time syndromic surveillance. International Journal of Environmental Research and Public Health. 2016;13(1).

72. Barbosa JV, Nunes RAO, Alvim-Ferraz MCM, Martins FG, Sousa SIV. Health and Economic Burden of the 2017 Portuguese Extreme Wildland Fires on Children. Int J Environ Res Public Health. 2022;19(1):593.

73. Vicedo-Cabrera AM, Esplugues A, Iñíguez C, Estarlich M, Ballester F. Health effects of the 2012 Valencia (Spain) wildfires on children in a cohort study. Environ Geochem Health. 2016;38(3):703–12.
